# Comparative effects of nebivolol and carvedilol on left ventricular diastolic function in older heart failure patients with preserved ejection fraction: study protocol for a randomized controlled trial

**DOI:** 10.1186/s13063-016-1656-y

**Published:** 2016-11-03

**Authors:** Kyungil Park, Tae-Ho Park

**Affiliations:** Division of Cardiology, Department of Internal Medicine, Dong-A University College of Medicine, 1 Dongdae-sin-dong 3-ga, Seo-gu, Busan, 602-715 South Korea

**Keywords:** Nebivolol, Carvedilol, Heart failure

## Abstract

**Background:**

Heart failure (HF) is a common and disabling condition in older people. Randomized clinical trials and meta-analyses have clearly demonstrated that the long-term use of β-Blockers improves the outcome of patients with HF. However, limited data are available on the treatment of older HF patients with preserved ejection fraction (EF). No study has specifically compared the relative effectiveness of carvedilol and nebivolol in treating HF in older patients with preserved EF.

**Method/design:**

This trial is a prospective, randomized, open-label, single-centre, active controlled study designed to investigate the effects of nebivolol and carvedilol on diastolic function of the left ventricle (LV) in older HF patients with preserved EF.

We will test the hypothesis that nebivolol improves LV diastolic function to a greater extent than carvedilol in patients over 70 years of age. The study population includes 62 older patients newly diagnosed with HF. Patients will be included in the study if they have a LVEF ≥40 %, New York Heart Association (NYHA) functional classes I, II or III status, and have been clinically stable without hospital admission for HF in the preceding 3 months. Eligible patients will be randomly assigned, in a 1:1 ratio, to receive a loading and maintenance dose of either nebivolol or carvedilol. Echocardiographic evaluations will be performed at baseline, 6, and 12 months after therapy. Clinical assessment and laboratory tests are to be performed at fixed times.

**Discussion:**

This trial is a single-center study that aims to evaluate the impact of nebivolol on LV diastolic function. The results of the study will provide information about the optimal choice of a β-Blocker in the management of patients after diagnosis of HF with preserved EF. The results will be available by 2017.

**Trial registration:**

ClinicalTrials.gov Identifier: NCT02619526, registered on 25 November 2015.

**Electronic supplementary material:**

The online version of this article (doi:10.1186/s13063-016-1656-y) contains supplementary material, which is available to authorized users.

## Background

Heart failure (HF) is a common disease in older people, and has a pronounced negative impact on quality of life and functional status [1, 2]. The use of β-Blockers has been proven to improve left ventricular (LV) systolic function and the prognoses of patients with HF in randomized controlled trials [3–6]. Thus, β-Blockers are considered a cornerstone in the pharmacological treatment of HF. However, data have shown that the median age of presentation of new HF cases is older than 75 years [2, 7, 8]. For patients with HF aged 70 and over the evidence for the efficacy of β-Blockers is limited. Only the SENIORS (Study of Effects of Nebivolol Intervention on Outcomes and Rehospitalization in Seniors With Heart Failure) trial has investigated the effect of nebivolol in HF patients over the age of 70 years. Those results demonstrated a significant reduction in the risk of death and cardiovascular-related hospitalization in patients given β-Blockers as compared with a placebo [6]. In a subanalysis of the SENIORS trial, the effect of nebivolol in older patients with HF was shown to be similar in those with preserved and impaired ejection fraction (EF) [9].

However, no study has specifically compared the relative effectiveness of nebivolol and carvedilol in treating HF in older patients with preserved LV function. This open-label study will compare the effects of long-term treatment with nebivolol versus carvedilol on LV diastolic function in older patients with HF.

## Trial objectives

The primary question to be tested is whether nebivolol improves LV diastolic function as measured by echocardiography in older patients with preserved EF during a follow-up period of 1 year, compared with the usual dose of carvedilol in the control group.

The secondary objective of the study is to assess the effect of the two β-Blockers on clinical outcomes. Clinical events are defined as symptom severity (New York Heart Association (NYHA) classification) and hospitalization due to HF.

## Methods/design

### Ethics

This study follows the principles set forth in the Helsinki Declaration, meaning that all patients sign a written informed consent stating that participation is voluntary and that participation can be withdrawn at any time, without any negative consequences concerning their current or future medical treatment. The study protocol (version #1, 7 October 2015), which follows the Standard Protocol Items: Recommendations for Interventional Trials (SPIRIT) guidelines (see Additional file 1), has been approved by the Institutional Review Board of Dong-A University Hospital.

### Patient population

This study will take place at Dong-A University Hospital. Patients will be eligible for the study if they have a left ventricular ejection fraction (LVEF) ≥40 %, NYHA functional classes I, II or III status, and have been clinically stable without hospital admission for HF in the preceding 3 months. Men and women over 70 years of age are eligible (Table 1). In the present study, preserved LVEF is defined as an EF ≥40 % using a modified Simpson’s rule [10]. Pharmacological therapy other than that with the study drug, including ACE and statin use, is allowed at the discretion of the attending physician. Patients who have contraindications to the study drug or major concomitant diseases are to be excluded (Table 1).

**Table 1:** Eligibility criteria

**Inclusion criteria** Subjects > 70 years of age Either gender First diagnosis of heart failure defined as: a) Mild or moderate symptomatic heart failure (NYHA calss I to III) b) An echocardiographic left ventricular ejection fraction ≥ 40% Patients who provide written informed consent
**Exclusion criteria** History and/or clinical documentation of pulmonary embolism Severe heart failure (NYHA IV or need for inotropic support) Primary valvular heart disease Pericardial disease Severe obstructive lung disease Primary pulmonary hypertension Occupational lung disease Asthma Severe renal failure (serum creatinine >2.0 mg/dL) Significant peripheral vascular disease Severe bradycardia (heart rate< 50 beats/minutes) Second or third-degree atrio-ventricular block Atrial fibrillation Life expectancy < 1 year Concern for inability of the patient to comply with study procedures and/or follow up Any condition which in the opinion of the Investigator would make it unsafe or unsuitable for the patient to participate in this study Involvement in the planning and/or conduct of the study Participation in another clinical study with an investigational product during the preceding 30 days Unable to give informed consent

### Study design

**Fig. 1:**
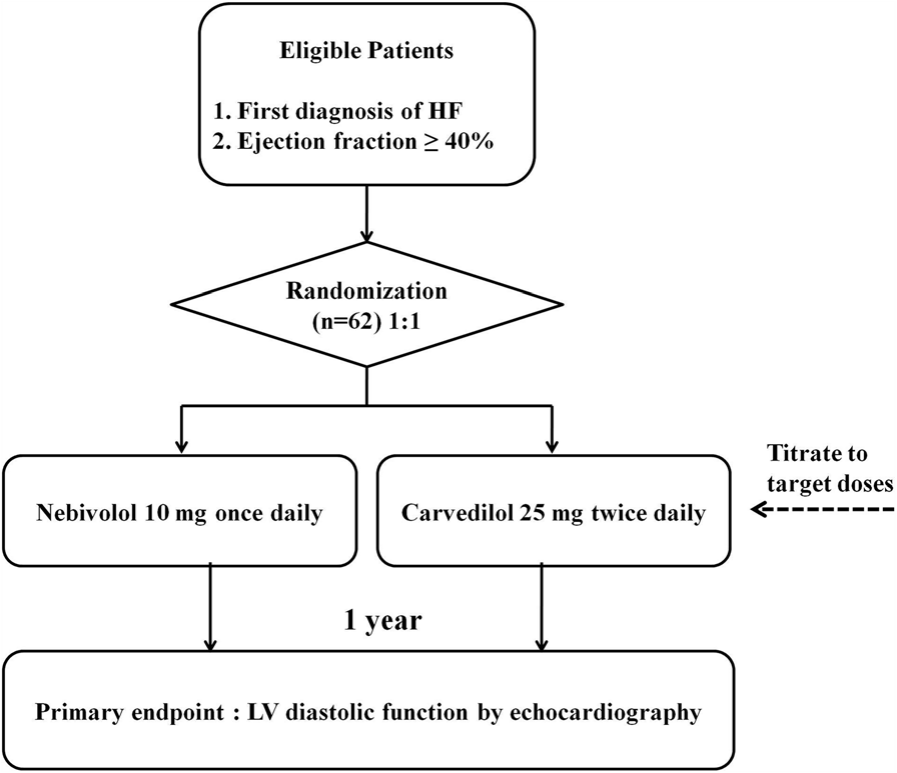
Overall algorithm of the study

This study is a prospective, randomized, open-label, single-centre, active controlled study with two parallel study groups. The choice of a prospective and open-label study was felt to be a reasonable reflection of clinical practice in only one center with a small sample. The randomized design was adopted to minimize bias in this single-center study. The dose of nebivolol was chosen on the basis of the SENIORS study [6]. The control arm will receive carvedilol because it is the β-Blocker that is indicated for the broadest population and is the most frequently used β-Blocker worldwide. A treatment duration of a minimum of 12 months was selected as a result of the SENIORS trial. The overall study design is depicted in Fig. 1.

### Randomization

All new patients admitted to the Dong-A University Cardiology Department will be screened for participation eligibility in the study by the principal investigator. Randomization following initial echocardiographic testing can be performed immediately after enrollment for patients who fulfill all the eligibility requirements. Patients will be randomly allocated to one of two conditions: (1) nebivolol treatment (10 mg once daily) or (2) carvedilol treatment (25 mg twice daily). Enrollment is expected to continue for approximately 12 months. Random treatment assignments will be generated using Excel spreadsheet software (Microsoft Corporation, Redmont, DC, USA). Eligible patients will be randomly assigned in a 1:1 ratio to receive either nebivolol or carvedilol. Randomization will be performed by an individual not involved in the study and will be kept concealed. Following randomization, the first dose of study medication will be administered to the patient as soon as possible. Potential attrition bias will be mitigated by only including patients who are motivated to recieve treatment. We will take efforts to minimize dropout and any potential biases attributable to dropout will be explored statistically. If a participant wishes to withdraw from the study, the reason for withdrawal will be documented in the participant’s records for the subsequent analysis in the interpretation of the results.

### Outcome measurements

The primary efficacy variables are mitral inflow velocities (E and A waves), deceleration time of the E wave (DT), isovolumetric relaxation time (IVRT), mitral annular velocities (early diastolic velocity of mitral annulus (Ea) and late diastolic velocity of mitral annulus (Aa) waves), left atrial (LA) volume, and LA strain (global systolic and diastolic LA strain), which will be measured via echocardiographic evaluation after 12 months of treatment. Mitral E/A and E/Ea ratios will be calculated. Parameters from Doppler analysis, M-mode echocardiography, and 2D-transthoracic echocardiography will be used. All echocardiographic images will be interpreted by an observer who is blinded to clinical data.

Secondary outcomes include occurrence of clinical events. Symptom severity (NYHA classification) and hospitalization will be evaluated at baseline and at maintenance follow-up visits. Vital signs, including heart rate and blood pressure, will be measured at every visit. Any complications, discontinuations, and deaths occurring during the study period will be noted. Each patient’s medical history and the results of a clinical examination will be recorded at baseline by the attending physician.

### Intervention and comparator descriptions

**Fig. 2:**
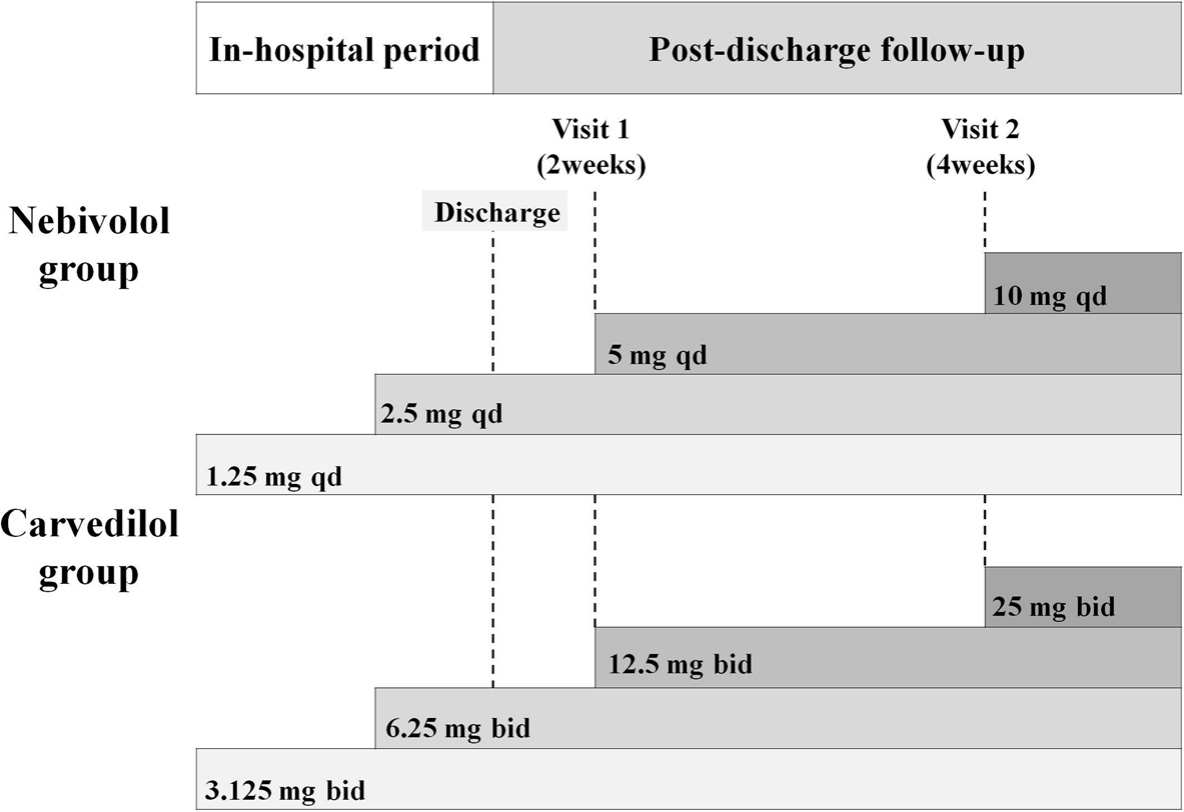
Titration scheme of the study drug

Patients will be followed with weekly visits during the up-titration phase. For nebivolol, the initial dose will be 1.25 mg once daily. If tolerated, it will be increased to 2.5 mg once daily by the end of week 1, 5 mg once daily by week 2 (visit 1), and 10 mg once daily by week 4 (visit 2). For carvedilol, the initial dose will be half of a 3.125-mg tablet twice daily. If tolerated, it will be increased to 6.25 mg twice daily by the end of week 1, 12.5 mg twice daily by week 2 (visit 1), and 25 mg twice daily by week 4 (visit 2). If side effects attributable to the study medications occur, up-titration will be delayed, the dose will be decreased, or the administration of the drug will be temporarily discontinued. If up-titration is not clinically feasible, either because of hypotension or bradycardia, the previous dose will be administered subsequently as the maximal tolerable dose. This process is illustrated in Fig. 2.

### Follow-up protocol

On achievement of the target dose or the maximum tolerable dose, patients will be followed up clinically every month for the first 3 months, then every 3 months until the end of the study at 12 months. At each visit, patients will undergo a complete physical examination, medical history-taking, and assessment of drug compliance. Investigators will evaluate all clinical and laboratory adverse events at each visit. NYHA functional class and predefined clinical events will be recorded at each clinical visit. Echocardiographic examinations will be performed at 6 and 12 months after discharge.

### Adverse effects

Analysis of safety-related data will be performed with respect to frequency of serious adverse events (SAE), stratified by causality and intensity of morbidity in both treatment groups. Patients will be interviewed at each visit about the occurrence of any adverse events, including the time of onset, duration, and severity; all information will be recorded on a Case Report Form. The causal relation to the study drug and the intensity of adverse events will be evaluated by the investigators. SAE must be reported to the Institutional Review Board and study sponsor by the principal investigator within 24 h after the SAE becomes known.

### Withdrawals

Patients are free to withdraw from participation in the trial at their own request at any time and without giving reasons for their decision. Moreover, the primary investigator can withdraw study patients if continuation of the trial would be detrimental to the patient’s wellbeing. Withdrawals will be documented in the Case Report Form and in the patient’s medical records and all ongoing SAE will be followed up.

### Sample size

Calculation of sample size was done based on the primary outcomes and the primary analysis for the intention-to-treat population. The sample size was calculated using the study’s primary objective, to detect a difference in E/E’ of 5.0 between nebivolol and carvedilol treatment with a power of 90 %. On the basis of previous reports, we assume the standard deviation of E/E’ to be approximately 5.0 in both treatment groups [11, 12]. We adjusted the sample size for an estimated follow-up loss rate of 30 %, a two-sided level of significance *a* = 5 % and a power of 1 − β = 90 %. Thirty-one patients in each group are required to detect statistical differences with a two-sided Student’s *t* test given these parameters. Therefore, a total of 62 patients will be included in the analysis. The statistical analyses will be performed on the full dataset, which will include data from all randomized patients receiving at least one dose of study medication. Patients who are judged to be ineligible after study registration will be excluded from the analysis. Given the sample size, this study is likely to be underpowered in terms of assessing secondary outcomes.

### Data collection, storage, and security

All paper files are to be stored in locked file cabinets, and electronic files are to be stored in password-protected, encrypted files. Both paper and electronic files will be accessed only by members of the research team. The final dataset will reside with the principal investigator.

### Statistical analyses

The intent-to-treat (ITT) analysis set will be used for efficacy analyses. All patients who have begun treatment will be included irrespective of their protocol adherence and continued participation in the study. Patients will be analyzed according to their randomized study medication irrespective of whether a SAE occurred before or following discontinuation of study medication. For baseline characteristics, continuous variables will be assessed using the Student’s *t* test and discrete variables will be compared using the chi-square test. The primary outcome will be compared using two-sample *t* tests. The two-sided null hypothesis for the primary outcome measure states that nebivolol and carvedilol lead to the same change in ventricular diastolic function during the 12 months after diagnosis. This null hypothesis will be tested by regression analysis adjusted for baseline values. Assessments for other echocardiographic indices will be compared between treatment groups after 12 months of follow-up using two-sample *t* tests. Analyses of changes will be performed using data from all patients for whom baseline and 12-month follow-up data are available. Event-free cumulative survival rates will be plotted using the Kaplan-Meier method and comparisons will be made between patients given nebivolol and carvedilol using the log-rank test. A Cox proportional hazards model, with the use of forward selection based on the likelihood ratio test, will be implemented for multivariate analysis to determine which prognostic factors identified in the univariate analysis, including mitral inflow velocities, DT, IVRT, mitral annular velocities, LA volume, and LA strain were significantly related to clinical events in the follow-up period.

A safety analysis dataset will be used to assess safety and tolerability variables. All patients who received at least one dose of nebivolol or carvedilol, and for whom post-dose data are available, will be included in the safety population. Patients will be censored 7 days after their last dose of study medication. A value of *p* < 0.05 will be considered statistically significant.

Potential attrition bias will be mitigated by conducting dropout analysis and intention-to-treat analysis. Given that dropouts are expected, multiple imputation, based on regression methods, will be performed to complete the data. A fully specified statistical analysis plan will be written before unmasking of the data.

The report presenting the primary findings from this study will follow the Consolidated Standards of Reporting Trials (CONSORT) 2010 guidelines. Study results will be disseminated to researchers and clinicians via publications and conference presentations. Authorship of published papers will follow established guidelines for defining the level of contribution that warrants authorship.

## Discussion

Aging represents both a convergence of declining cardioprotective systems and increasing disease processes that are fertile ground for the development of HF [2]. With 50 % of all HF diagnoses and 90 % of all HF deaths occurring in the segment of the population over age 70, HF is largely a disease of older people. In addition, the greatest proportion of older HF patients have preserved systolic function [1].

Patients with HF can be divided into two different groups based on EF: HF with preserved or reduced EF [13]. The two are similar in terms of prevalence and mortality rate [14, 15]. However, current HF guidelines do not address the use of β-Blockers in patients with preserved EF [16] because no effective treatment has been identified in randomized clinical trials.

β-Blockers are thought to potentially improve diastolic filling by a negative chronotropic effect [17]. Some studies support the use of β-Blockers in patients with preserved EF [18, 19]. Notably, recent data suggest that the effect of the β-Blocker nebivolol is similar in HF patients with preserved and impaired EF [9]. Increased nitric oxide release induced by nebivolol [20] may encourage early LV relaxation [21].

Randomized clinical trials and meta-analyses have shown that long-term use of β-Blockers improves LV systolic function and clinical outcomes of HF patients with reduced EF. The average age of subjects in these placebo-controlled mortality and morbidity studies of β-Blockers in HF has been below 70 years [3–5]. Several β-Blockers are available, but only four, carvedilol, metoprolol, bisoprolol, and nebivolol, have been successfully tested in HF and are used in treatment of the condition [22, 23]. However, comparison studies on the efficiency of different β-Blockers in the treatment of HF are limited [23, 24]. At present, little data are available on the comparative effectiveness of carvedilol and nebivolol for HF. A recent study found that carvedilol and nebivolol appear to similarly improve LV diastolic functions in nonischemic HF patients [25]. However, that study did not involve an HF population with preserved EF. Thus, the current trial will be the first head-to-head comparison of the diastolic effects of nebivolol and carvedilol in HF patients with preserved EF.

With regard to possible risks of bias, we anticipate that the risk of selection bias is low, due to the use of adequate methods for sequence generation and allocation sequence concealment. Regarding performance, participants might be influenced by knowledge of group assignment; however, the possibility that unblinding to group assignment may result in an overestimation or an underestimation of intervention effects is low. In addition, as reported above, the risk of attrition bias due to missing data has been adequately prevented and will be treated.

## Trial status

Recruitment is ongoing at the time of manuscript submission.

## Additional file

Additional file 1: Appendix A. SPIRIT 2013 checklist: recommended items to address in a clinical trial protocol and related documents*. **Appendix B.** World Health Organization Trial Registration Dataset. **Appendix C.** Informed consent (only for Korean participants. (DOC 182 kb)

## Abbreviations

ACE: Angiotensin-converting enzyme
EF: Ejection fraction
HF: Heart failure
LV: Left ventricle
NYHA: New York Heart Association
SENIOR: Study of the Effects of Nebivolol Intervention on Outcomes and Rehospitalization in Seniors with Heart Failure
