# Supplementary material for: Comparative effects of nebivolol and carvedilol on left ventricular diastolic function in older heart failure patients with preserved ejection fraction: study protocol for a randomized controlled trial
Source: Trials. 2016 Nov 3;17:530. doi: 10.1186/s13063-016-1656-y (PMC5093969; doi:10.1186/s13063-016-1656-y)
Supplement: Additional file 1: Appendix A. — SPIRIT 2013 checklist: recommended items to address in a clinical trial protocol and related documents*. Appendix B. World Health Organization Trial Registration Dataset. Appendix C. Informed consent (only for Korean participants. (DOC 182 kb) [file 13063_2016_1656_MOESM1_ESM.doc]

**Appendix A: SPIRIT 2013 Checklist: Recommended items to address in a clinical trial protocol and related documents***


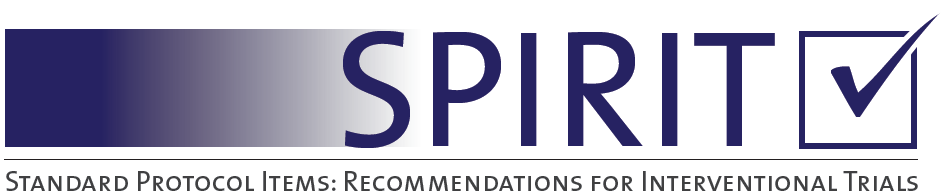


| Section/item | Item No | Description | Addressed on page number |
| --- | --- | --- | --- |
| **Administrative information** | | |  |
| Title | 1 | Descriptive title identifying the study design, population, interventions, and, if applicable, trial acronym | 1 |
| Trial registration | 2a | Trial identifier and registry name. If not yet registered, name of intended registry | 3 |
|  | 2b | All items from the World Health Organization Trial Registration Data Set | Appendix B |
| Protocol version | 3 | Date and version identifier | 5 |
| Funding | 4 | Sources and types of financial, material, and other support | 14 |
| Roles and responsibilities | 5a | Names, affiliations, and roles of protocol contributors | 1, 14 |
|  | 5b | Name and contact information for the trial sponsor | 1 |
|  | 5c | Role of study sponsor and funders, if any, in study design; collection, management, analysis, and interpretation of data; writing of the report; and the decision to submit the report for publication, including whether they will have ultimate authority over any of these activities | 11, 14 |
|  | 5d | Composition, roles, and responsibilities of the coordinating centre, steering committee, endpoint adjudication committee, data management team, and other individuals or groups overseeing the trial, if applicable (see Item 21a for data monitoring committee) | NA |
| Introduction |  |  |  |
| Background and rationale | 6a | Description of research question and justification for undertaking the trial, including summary of relevant studies (published and unpublished) examining benefits and harms for each intervention | 4 |
|  | 6b | Explanation for choice of comparators | 6 |
| Objectives | 7 | Specific objectives or hypotheses | 5 |
| Trial design | 8 | Description of trial design including type of trial (eg, parallel group, crossover, factorial, single group), allocation ratio, and framework (eg, superiority, equivalence, noninferiority, exploratory) | 6 |
| Methods: Participants, interventions, and outcomes | | |  |
| Study setting | 9 | Description of study settings (eg, community clinic, academic hospital) and list of countries where data will be collected. Reference to where list of study sites can be obtained | 5 |
| Eligibility criteria | 10 | Inclusion and exclusion criteria for participants. If applicable, eligibility criteria for study centres and individuals who will perform the interventions (eg, surgeons, psychotherapists) | 5, Table 1 |
| Interventions | 11a | Interventions for each group with sufficient detail to allow replication, including how and when they will be administered | 7-8 |
|  | 11b | Criteria for discontinuing or modifying allocated interventions for a given trial participant (eg, drug dose change in response to harms, participant request, or improving/worsening disease) | 9 |
|  | 11c | Strategies to improve adherence to intervention protocols, and any procedures for monitoring adherence (eg, drug tablet return, laboratory tests) | 6-7 |
|  | 11d | Relevant concomitant care and interventions that are permitted or prohibited during the trial | 6 |
| Outcomes | 12 | Primary, secondary, and other outcomes, including the specific measurement variable (eg, systolic blood pressure), analysis metric (eg, change from baseline, final value, time to event), method of aggregation (eg, median, proportion), and time point for each outcome. Explanation of the clinical relevance of chosen efficacy and harm outcomes is strongly recommended | 7-8 |
| Participant timeline | 13 | Time schedule of enrolment, interventions (including any run-ins and washouts), assessments, and visits for participants. A schematic diagram is highly recommended (see Figure) | 8, Figure 1-2 |
| Sample size | 14 | Estimated number of participants needed to achieve study objectives and how it was determined, including clinical and statistical assumptions supporting any sample size calculations | 9-10 |
| Recruitment | 15 | Strategies for achieving adequate participant enrolment to reach target sample size | 6 |

| **Methods: Assignment of interventions (for controlled trials)** | | |  |
| --- | --- | --- | --- |
| Allocation: |  |  |  |
| Sequence generation | 16a | Method of generating the allocation sequence (eg, computer-generated random numbers), and list of any factors for stratification. To reduce predictability of a random sequence, details of any planned restriction (eg, blocking) should be provided in a separate document that is unavailable to those who enrol participants or assign interventions | 6 |
| Allocation concealment mechanism | 16b | Mechanism of implementing the allocation sequence (eg, central telephone; sequentially numbered, opaque, sealed envelopes), describing any steps to conceal the sequence until interventions are assigned | 7 |
| Implementation | 16c | Who will generate the allocation sequence, who will enrol participants, and who will assign participants to interventions | 6 |
| Blinding (masking) | 17a | Who will be blinded after assignment to interventions (eg, trial participants, care providers, outcome assessors, data analysts), and how | NA |
|  | 17b | If blinded, circumstances under which unblinding is permissible, and procedure for revealing a participant’s allocated intervention during the trial | NA |
| **Methods: Data collection, management, and analysis** | | |  |
| Data collection methods | 18a | Plans for assessment and collection of outcome, baseline, and other trial data, including any related processes to promote data quality (eg, duplicate measurements, training of assessors) and a description of study instruments (eg, questionnaires, laboratory tests) along with their reliability and validity, if known. Reference to where data collection forms can be found, if not in the protocol | 7 |
|  | 18b | Plans to promote participant retention and complete follow-up, including list of any outcome data to be collected for participants who discontinue or deviate from intervention protocols | 6-7 |
| Data management | 19 | Plans for data entry, coding, security, and storage, including any related processes to promote data quality (eg, double data entry; range checks for data values). Reference to where details of data management procedures can be found, if not in the protocol | 10 |
| Statistical methods | 20a | Statistical methods for analysing primary and secondary outcomes. Reference to where other details of the statistical analysis plan can be found, if not in the protocol | 10-11 |
|  | 20b | Methods for any additional analyses (eg, subgroup and adjusted analyses) | 11 |
|  | 20c | Definition of analysis population relating to protocol non-adherence (eg, as randomised analysis), and any statistical methods to handle missing data (eg, multiple imputation) | 11 |
| **Methods: Monitoring** | | |  |
| Data monitoring | 21a | Composition of data monitoring committee (DMC); summary of its role and reporting structure; statement of whether it is independent from the sponsor and competing interests; and reference to where further details about its charter can be found, if not in the protocol. Alternatively, an explanation of why a DMC is not needed | NA |
|  | 21b | Description of any interim analyses and stopping guidelines, including who will have access to these interim results and make the final decision to terminate the trial | NA |
| Harms | 22 | Plans for collecting, assessing, reporting, and managing solicited and spontaneously reported adverse events and other unintended effects of trial interventions or trial conduct | 8-9 |
| Auditing | 23 | Frequency and procedures for auditing trial conduct, if any, and whether the process will be independent from investigators and the sponsor | NA |
| Ethics and dissemination | | |  |
| Research ethics approval | 24 | Plans for seeking research ethics committee/institutional review board (REC/IRB) approval | 5 |
| Protocol amendments | 25 | Plans for communicating important protocol modifications (eg, changes to eligibility criteria, outcomes, analyses) to relevant parties (eg, investigators, REC/IRBs, trial participants, trial registries, journals, regulators) | NA |
| Consent or assent | 26a | Who will obtain informed consent or assent from potential trial participants or authorised surrogates, and how (see Item 32) | 6 |
|  | 26b | Additional consent provisions for collection and use of participant data and biological specimens in ancillary studies, if applicable | NA |
| Confidentiality | 27 | How personal information about potential and enrolled participants will be collected, shared, and maintained in order to protect confidentiality before, during, and after the trial | 10 |
| Declaration of interests | 28 | Financial and other competing interests for principal investigators for the overall trial and each study site | 13 |
| Access to data | 29 | Statement of who will have access to the final trial dataset, and disclosure of contractual agreements that limit such access for investigators | 10 |
| Ancillary and post-trial care | 30 | Provisions, if any, for ancillary and post-trial care, and for compensation to those who suffer harm from trial participation | NA |
| Dissemination policy | 31a | Plans for investigators and sponsor to communicate trial results to participants, healthcare professionals, the public, and other relevant groups (eg, via publication, reporting in results databases, or other data sharing arrangements), including any publication restrictions | 10,11 |
|  | 31b | Authorship eligibility guidelines and any intended use of professional writers | 11 |
|  | 31c | Plans, if any, for granting public access to the full protocol, participant-level dataset, and statistical code | NA |
| Appendices |  |  |  |
| Informed consent materials | 32 | Model consent form and other related documentation given to participants and authorised surrogates | Appendix C |
| Biological specimens | 33 | Plans for collection, laboratory evaluation, and storage of biological specimens for genetic or molecular analysis in the current trial and for future use in ancillary studies, if applicable | NA |

*It is strongly recommended that this checklist be read in conjunction with the SPIRIT 2013 Explanation & Elaboration for important clarification on the items. Amendments to the protocol should be tracked and dated. The SPIRIT checklist is copyrighted by the SPIRIT Group under the Creative Commons “[Attribution-NonCommercial-NoDerivs 3.0 Unported](http://www.creativecommons.org/licenses/by-nc-nd/3.0/)” licens

**Appendix B: World Health Organization Trial Registration Data Set**

1. **Primary Registry and Trial Identifying Number:** ClinicalTrials.gov; NCT02619526; URL https://clinicaltrials.gov/ct2/show/NCT02619526.
2. **Date of Registration in Primary Registry:** November 25, 2015
3. **Secondary Identifying Numbers:** NA.
4. **Source(s) of Monetary or Material Support:** Menarini Korea, 12F SungDam Glag., 411, Teheran-ro Gangnam-gu, Seoul, Korea; Phone +82220377300; Fax: +82220377373
5. **Primary Sponsor:** Dong-A University hospital, 1 Dongdae-sin-dong 3-ga, Seo-gu, Busan 602-715, Korea; Phone +82512402059; Fax: +82512402044; e-mail: [info@metabolicfitness.it](mailto:info@metabolicfitness.it).
6. **Secondary Sponsor(s):** NA.
7. **Contact for Public Queries:** Kyungil Park, MD, Division of Cardiology, Department of Internal Medicine, Dong-A University College of Medicine, 1 Dongdae-sin-dong 3-ga, Seo-gu, Busan 602-715, Korea; Phone: +82-512402059; Fax: +82-512402044; e-mail: cardiopark@gmail.com.
8. **Contact for Scientific Queries:** Tae-Ho Park, MD, PhD Professor, Division of Cardiology, Department of Internal Medicine, Dong-A University College of Medicine, 1 Dongdae-sin-dong 3-ga, Seo-gu, Busan 602-715, Korea; Phone: +82-512402059; Fax: +82-512402044; E-mail: thpark65@dau.ac.kr.
9. **Public Title:** Comparative Effects of Nebivolol and Carvedilol on Diastolic Function in the Elderly Heart Failure Patients
10. **Scientific Title:** Comparative effects of nebivolol and carvedilol on diastolic function of the left ventricle in the elderly heart failure patients with preserved ejection fraction: study protocol for a randomized controlled trial
11. **Countries of Recruitment:** Korea.
12. **Health Condition(s) or Problem(s) Studied:** Heart failure.
13. **Intervention:**
14. Intervention arm

- Name: Treatment with Nebivolol.
- Description: nebivolol treatment (10 mg once daily)

1. Control arm: Treatment with Carvedilol.
2. **Key Inclusion and Exclusion Criteria**
3. Inclusion criteria: First diagnosis of heart failure. Additional requirements are age above 70 years.
4. Exclusion criteria: History and/or clinical documentation of pulmonary embolism, Severe heart failure (NYHA IV or need for inotropic support), Primary valvular heart disease, Pericardial disease, Severe obstructive lung disease, Primary pulmonary hypertension, Occupational lung disease, Asthma, Severe renal failure (serum creatinine >2.0 mg/dL), Significant peripheral vascular disease, Severe bradycardia (heart rate< 50 beats/minutes), Second or third-degree atrio-ventricular block, Atrial fibrillation, Life expectancy < 1 year, Concern for inability of the patient to comply with study procedures and/or follow up, Any condition which in the opinion of the Investigator would make it unsafe or unsuitable for the patient to participate in this study, Involvement in the planning and/or conduct of the study, Participation in another clinical study with an investigational product during the preceding 30 days, Unable to give informed consent
5. **Study Type**
   1. Type of study: interventional.
   2. Study design:
      - Method of allocation: randomized
      - Masking: no
      - Assignment: parallel
      - Purpose: investigating effects of nebivolol and carvedilol on diastolic function of the left ventricle (LV) in the elderly HF patients with preserved EF
6. Phase: NA
7. Allocation concealment mechanism and sequence generation: Eligible patients are randomly assigned in a 1:1 ratio to receive nebivolol or carvedilol. Random treatment assignments will be generated using Excel spreadsheet software.
8. **Date of First Enrollment:** April 7, 2016.
9. **Target Sample Size:** 62
10. **Recruitment Status: recruiting.**
11. **Primary Outcome(s)**
    - Name: diastolic function
    - Method of measurement: Doppler analysis of echocardiogram
    - Time points: 12 months.
12. **Key Secondary Outcomes**
    1. Name: occurrence of clinical events: every 3 months for 12 months.

**Appendix C: Informed consent (only for Korean)**

**피험자용 설명 및 동의서**

**연구 과제명**: 이완기 심부전 환자의 좌심실 이완 기능 회복에 대한 베타차단제 비교 전향적 무작위 연구

**연구 책임자명**: 동아대학교 병원 순환기내과 교수 박 태 호

1. **본 임상시험은 연구목적으로 수행됩니다.**

본 임상연구의 목적은 이완기 심부전 환자의 치료에서 베타 차단제인 네비볼롤(nebivolol)투여가 카베딜롤(carvedilol)과 비교해서 좌심실 이완 기능 회복 효과가 있는가를 알아보고자 하는 비교 연구입니다.

1. **연구방법 및 예측 효능, 효과**

이완기 심부전은 고령에서 흔히 관찰하게 되는 질환이며, 좌심실 이완 기능이 저하된 환자일수록 사망률의 위험도가 높은 것으로 알려져 있습니다. 따라서 좌심실 이완 기능이 저하된 환자에서 심부전 약제 사용은 더욱 필요하며, 사망률을 줄이기 위해 다양한 약제가 개발 연구되고 있습니다. 그러나 수축기 심부전과 달리 이완기 심부전의 치료 약제는 아직 치료지침이 정리가 되어 있지 않습니다. 베타 차단제 및 칼슘차단제 는 혈압 및 후부하를 감소시키고 이완기 충만시간을 증가시켜 이상적인 약으로 여겨졌으나, 생존율을 증가시키지는 못하였습니다.

네비볼를은 최근에 개발된 베타 차단제로 좌심실 기능이 저하된 심부전 환자에서 사망률 개선과 증상 개선을 위해 처방되고 있는 약제입니다. 특히, 70세 이상의 노인 환자에게 네비볼롤을 투여하였을 때 사망률의 현저한 개선을 보여, 노인 환자에게 특히 효과가 있을 것으로 기대되고 있습니다. 이에 본 연구에서는 이완기 심부전 환자에서 네비볼롤 투여가 좌심실 이완 기능 회복에 미치는 영향에 대해 규명하고자 합니다.

1. **본 임상시험의 선정기준**

숨이 찬 증상이 있거나 양쪽 다리가 붓는 증상이 있으면서, 심장 초음파 검사에서 좌심실 이완기 기능이 장애가 발생한 환자가 본 연구에 참여할 수 있습니다.

1. **시험에 사용되는 약물**

본 연구에서 사용되는 연구 약제인 네비볼롤과 카베딜롤은 베타 차단제로서, 심부전에서 이미 그 안전성과 유용성이 입증되어 국내를 포함한 여러 국가의 임상에서 널리 이용되고 있습니다. 그리고 본 연구에 참여하는 모든 피험자는 현재 사용되고 있는 이완기 심부전의 모든 표준 치료법에 따라 치료를 유지하기 때문에 피험자가 이 연구에 참여함으로써 발생하는 추가 위험성은 없을 것으로 판단됩니다.

1. **본 연구에 사용되는 기구**

본 연구에서 새로이 사용되는 기구는 없습니다.

1. **본 연구에 참여하게 됨으로써 받게 되는 검사 및 절차**

본 연구에 참여하시게 됨으로써 추가적으로 심장초음파 검사를 받게 됩니다. 심장초음파 검사는 이완기 심부전의 진단을 위해 흔히 사용되는 검사법이며, 검사 방법이 비침습적으로 매우 안전합니다. 이 외 임상 연구에 참여하지 않는 다른 환자분들과 비교해서 치료 및 절차는 모두 동일합니다.

1. **본 연구를 위해서 피험자가 준수해야 하는 사항**

본 연구에 참여하시는 환자분이 준수해야 하는 사항은 임상연구에 참여하지 환자와 같습니다.

1. **본 임상시험의 검증되지 않은 실험적인 측면**

해당 사항 없습니다.

1. **임상시험에 참여함으로써 피험자에게 미칠 것으로 예견되는 위험(부작용)이나 불편사항**

해당 사항 없습니다.

1. **이 임상시험에 참여함으로써 기대되는 이익**

참여 환자를 대상으로 심장 초음파 검사가 시행되는데 해당 검사를 무상으로 제공받게 됩니다.

1. **본 질환으로 선택할 수 있는 다른 치료방법 및 이러한 치료의 잠재적 위험과 이익**

참여 환자는 심부전 치료의 표준 요법에 따라 치료됩니다. 주치의가 판단하여 이 외의 치료방법이 환자에게 더 큰 이득을 가져올 것으로 예상되는 경우에는 상기 연구에 참여하지 않게 될 것입니다.

1. **예상 참여기간 및 본 시험에 참여하는 대략의 전체 피험자수**

이완기 심부전으로 진단된 후 12개월까지이며 64명이 이 연구에 참여하게 됩니다. 64명의 참여자는 무작위 배정에 의해 32명은 네비볼롤를 복용하고, 32명은 카베딜롤을 복용하게 됩니다.

1. **임상시험과 관련된 손상이 발생하였을 경우 피험자에게 주어질 보상이나 치료 방법**

예상치 못한 이상 반응이 발생할 경우, 신속하게 해당 분야의 전문의에게 진료를 받도록 하여 조기에 이상 반응이 치료될 수 있게 하며 환자의 부담이 최소화되도록 노력할 것입니다. 치료 도중에 발생하는 어떠한 종류의 부작용에 대하여도 세심하게 관찰이 기울여질 것이며 부작용이 발생하면 본 의료진 전원에게 알려지고, 가장 좋은 치료가 수행되도록 할 것입니다. 따라서 치료 도중이나 후 어떠한 이상이라도 발견되면 의료진에게 언제든지 문의하시기 바랍니다.

1. **임상시험에 참여함으로써 받게 되는 금전적 보상의 여부 및 참여 정도에 따른 조정 정도 또는 임상시험에 참여함으로써 피험자에게 추가적으로 발생이 예상되는 비용**

본 연구에 참여하시더라도 경제적인 보상은 없습니다. 또한 본 연구에 참여하시더라도 추가적으로 발생되는 비용은 없습니다.

1. **다음의 경우에는 보상하지 않습니다.**

1) 연구책임자의 후원 하에 시행되지 않았거나 연구자가 제공하지 않은 의약품 등으로 인하여 발생한 이상반응의 경우

2) 서로 합의한 임상시험계획서를 준수하지 않아 일어난 손상(임상시험 계획서에 요구되지 않은 검사 또는 치료적 조치로 인한 경우 포함)

3) 피험자의 부주의로부터 발생된 경우

4) 환자의 기저 질환의 진행 및 악화에 의해 발생한 경우

5) 심부전 치료 중 발생할 수 있는 일반적인 부작용

6) 질병의 자연 경과에 의해 발생한 손상

1. **본 임상연구에서 기타 고려사항은 다음과 같습니다.**

**피험자가 받게 되는 새로운 정보**: 임상연구 지속 참여 의지에 영향을 줄 수 있는 새로운 정보가 얻어지면 적시에 본인 또는 대리인에게 알려집니다. 본 연구진행 중 본인에게 영향을 줄 수도 있는 새로운 정보를 연구자가 획득하게 되면 그 내용을 통보 받을 수 있습니다.

**임상연구 참여의 제한**: 연구책임자가 필요하다고 판단 될 경우 본인의 동의 없이도 본 연구 참여에서 제한될 수 있습니다. 또한, 이 경우 연구책임자 또는 동의서상에 명시된 연구자에게 통보 함으로써 본 연구에 불참할 수 있습니다.

**자유의사에 따른 임상연구에의 참여**: 환자분이 이 임상연구에 참여해야 할 의무는 없으며, 참여 여부는 본인 자유의사에 의하여 결정됩니다. 동의서에 서명하고 임상연구에 참여한 후에도 언제나 환자분이 원하면 이를 중단할 수 있습니다. 그렇더라도 본 병원에서 계속 치료 받는데 있어서 불이익이나 다른 환자와의 차별은 일체 없습니다.

**비밀 보장**: 모니터요원, 점검을 실시하는 자, 심사위원회 및 식품의약품안전청장은 피험자의 비밀보장을 침해하지 않고 관련규정이 정하는 범위 안에서 임상연구의 실시절차와 자료의 신뢰성을 검증하기 위해 본인의 의무기록을 직접 열람할 수 있습니다. 본 동의서에 서명함은 이러한 자료의 직접 열람을 허용한다는 것을 의미합니다. 본 임상연구의 결과가 출판될 경우 피험자의 신원은 비밀 상태로 유지됩니다.

본 연구에 대해 질문이 있거나 연구 중간에 문제가 생길 시 다음 연구 담당자에게 연락하십시오.

이름: 이 은 지 전화번호 051-240-2959

만일 어느 때라도 피험자로서 귀하의 권리에 대한 질문이 있다면 다음의 동아대학교 병원 임상연구심의위원회로 연락하십시오.

동아대학교 병원 임상연구심의위원회 전화번호: 051-240-2611 (동아대학교병원)

**동 의 서**

1. 나는 이 설명서를 읽었으며 담당 연구원과 이에 대하여 의논하였습니다.
2. 나는 위험과 이득에 관하여 들었으며 나의 질문에 만족할 만한 답변을 얻었습니다.
3. 나는 이 연구에서 얻어진 나에 대한 정보를 현행 법률과 임상연구심의위원회 규정이 허용하는 범위 내에서 연구자가 수집하고 처리하는데 동의합니다.
4. 나는 이 연구에 참여하는 것에 대하여 자발적으로 동의합니다.
5. 나는 담당 연구자나 위임 받은 대리인이 연구를 진행하거나 결과 관리를 하는 경우와 보건 당국, 학교 당국 및 동아대학교 병원 임상연구심의위원회가 실태 조사를 하는 경우에는 비밀로 유지되는 나의 개인 신상 정보를 직접적으로 열람하는 것에 동의합니다.
6. 나는 언제라도 이 연구의 참여를 철회할 수 있고 이러한 결정이 나에게 어떠한 해도 되지 않을 것이라는 것을 압니다.
7. 나의 서명은 이 동의서의 사본을 받았다는 것을 뜻하며 연구 참여가 끝날 때까지 사본을 보관하겠습니다.

| 피험자 성명 |  | 서명 |  | 날짜 (년/월/일) |  |
| --- | --- | --- | --- | --- | --- |
| 동의서 받은 연구원 성명 |  | 서명 |  | 날짜 (년/월/일) |  |
| 연구책임자 성명 |  | 서명 |  | 날짜 (년/월/일) |  |
| 만일 있을 경우 | | | | | |
| 법적 대리인 성명 |  | 서명 |  | 날짜 (년/월/일) |  |
| 입회인 성명 |  | 서명 |  | 날짜 (년/월/일) |  |
